# IL-4 and IL-13 exposure during mucociliary differentiation of bronchial epithelial cells increases antimicrobial activity and expression of antimicrobial peptides

**DOI:** 10.1186/1465-9921-12-59

**Published:** 2011-05-02

**Authors:** Suzanne Zuyderduyn, Dennis K Ninaber, Jasmijn A Schrumpf, Marianne AJA van Sterkenburg, Renate M Verhoosel, Frans A Prins, Sandra van Wetering, Klaus F Rabe, Pieter S Hiemstra

**Affiliations:** 1 Department of Pulmonology, Leiden University Medical Center, Leiden, The Netherlands; 2 Pathology, Leiden University Medical Center, Leiden, The Netherlands; 3 DCPrime BV, De Boelelaan 1085, 1081 HV, Amsterdam, The Netherlands

**Keywords:** human, lung, cell differentiation, allergy, inflammation

## Abstract

The airway epithelium forms a barrier against infection but also produces antimicrobial peptides (AMPs) and other inflammatory mediators to activate the immune system. It has been shown that in allergic disorders, Th2 cytokines may hamper the antimicrobial activity of the epithelium. However, the presence of Th2 cytokines also affects the composition of the epithelial layer which may alter its function. Therefore, we investigated whether exposure of human primary bronchial epithelial cells (PBEC) to Th2 cytokines during mucociliary differentiation affects expression of the human cathelicidin antimicrobial protein (hCAP18)/LL-37 and human beta defensins (hBD), and antimicrobial activity.

PBEC were cultured at an air-liquid interface (ALI) for two weeks in the presence of various concentrations of IL-4 or IL-13. Changes in differentiation and in expression of various AMPs and the antimicrobial proteinase inhibitors secretory leukocyte protease inhibitor (SLPI) and elafin were investigated as well as antimicrobial activity.

IL-4 and IL-13 increased mRNA expression of hCAP18/LL-37 and hBD-2. Dot blot analysis also showed an increase in hCAP18/LL-37 protein in apical washes of IL-4-treated ALI cultures, whereas Western Blot analysis showed expression of a protein of approximately 4.5 kDa in basal medium of IL-4-treated cultures. Using sandwich ELISA we found that also hBD-2 in apical washes was increased by both IL-4 and IL-13. SLPI and elafin levels were not affected by IL-4 or IL-13 at the mRNA or protein level. Apical wash obtained from IL-4- and IL-13-treated cultures displayed increased antimicrobial activity against *Pseudomonas aeruginosa *compared to medium-treated cultures. In addition, differentiation in the presence of Th2 cytokines resulted in increased MUC5AC production as has been shown previously.

These data suggest that prolonged exposure to Th2 cytokines during mucociliary differentiation contributes to antimicrobial defence by increasing the expression and release of selected antimicrobial peptides and mucus.

## Background

The airway epithelium is a pseudostratified columnar epithelium containing basal, secretory and ciliated cells. This layer constantly regenerates through migration, proliferation and differentiation of epithelial cells to form a barrier to protect against inhaled pathogens. In addition to its barrier function, the epithelium provides mucociliary clearance and releases a variety of mediators such as antimicrobial peptides (AMPs; e.g. the human cathelicidin LL-37 and human beta-defensins [hBD]) and cytokines like the chemokine CXCL8 (interleukin [IL]-8). These mediators initiate and regulate the inflammatory response by inducing recruitment of phagocytes such as neutrophils and monocytes. Due to the influx of these cells and their released compounds local tissue injury occurs. To counteract this injury, the airway epithelium secretes serine proteinase inhibitors such as secretory leukocyte proteinase inhibitor (SLPI) and elafin [1,2], which also display antimicrobial activity *in vitro *against bacteria, fungi and certain viruses (e.g. HIV) [3-6].

Th2 cytokines are expressed in the airways of asthmatics [7,8]. Various studies including our own [9] have indicated that Th2 cytokines are able to influence the phenotype of the airway epithelium. Animal models have shown that Th2 cytokines such as IL-13 induce goblet cell hyperplasia [10] and *in vitro *studies of epithelial cell cultures have shown that the presence of IL-13 during mucociliary differentiation increases goblet cell hyperplasia [11], increases mRNA expression of mucins, decreases mRNA expression of ciliated cell markers FOXJ1, tektin and the novel marker ciliated bronchial epithelial-1 (CBE-1) [12], and increases MUC5AC protein expression [13]. Likewise, it was shown that also IL-4 can drive differentiation of cultured human airway epithelial cells towards a mucus hypersecretory phenotype [13].

We have previously shown that differentiation of airway epithelium markedly affects its function as squamous differentiation of PBEC results in release of more eotaxin-2/CCL24, whereas mucociliary differentiated PBEC (due to the presence of high concentrations of retinoic acid) release more eotaxin-3/CCL26 [9]. In that study [9], we have also shown that presence of IL-4 (or IL-13 to a lesser extent) during the differentiation phase resulted in increased expression of these eotaxins, as well as in an altered epithelial layer.

Antimicrobial defence is thought to be reduced in Th2 driven diseases such as asthma and atopic dermatitis (AD). Studies in atopic dermatitis (AD) patients have shown that the expression of antimicrobial peptides (LL-37, hBD-2 and -3) and proteinase inhibitors (SLPI and elafin) in skin is reduced compared to psoriatic skin. This could explain the increased susceptibility of AD patients to skin infection [14,15]. Since AD is a Th2 driven disease, these data suggest that the Th2 cytokine milieu may be detrimental for the antimicrobial defence provided by epithelial cells. Indeed, in cultured keratinocytes, IL-4 and IL-13 reduce the TNF-α/IFN-γ-induced hBD-2 and hBD-3 expression [16]. In addition, IL-13 was shown to reduce LL-37 expression in keratinocytes [17]. Not only in skin, but also in the airways Th2 cytokines may affect antimicrobial defence both *in vitro *and *in vivo *[18]. First, in cell culture experiments Th2 cytokines reduced the ability of differentiated human bronchial epithelial cells to clear *Pseudomonas aeruginosa *and the associated increase in hBD-2 mRNA expression. Second, inducing allergic airways inflammation in mice resulted in decreased pulmonary clearance of *P. aeruginosa *and a decrease in CRAMP, the murine homologue of hCAP18/LL-37, in lung lavage fluid [18].

In asthma the constantly regenerating epithelium is exposed to Th2 cytokines during differentiation rather than after differentiation, which leads to goblet cell hyperplasia and increased mucus production, but may also affect other functions of the epithelial layer such as antimicrobial defence. Therefore, in the present study we investigated the effect of presence of Th2 cytokines during mucociliary differentiation on host defence, in particular expression of the antimicrobial peptides LL-37, hBD-2, and -3, and the antimicrobial proteinase inhibitors SLPI and elafin. In addition, we investigated effects of Th2 cytokine-induced differentiation on morphology and antimicrobial activity of our cultures.

## Materials and methods

### Culture of human airway epithelial cells

**Figure 1:**
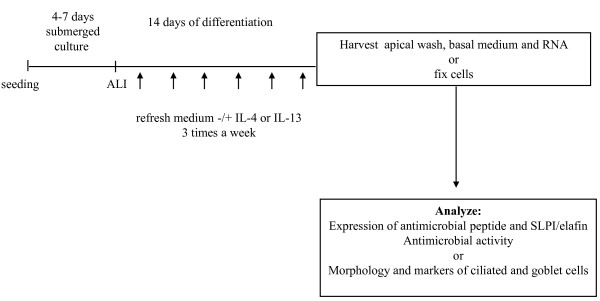
**Schematic representation of the air-liquid interface (ALI) culture model**. Primary bronchial epithelial cells were seeded onto transwell filters and allowed to grow to confluence for 4-7 days. Cells were exposed to air at the apical side (air-liquid interface; ALI) and stimulated for 14 days at the basal side with medium (containing high concentrations of retinoic acid to induce mucociliary differentiation). To investigate effects of IL-4 or IL-13 on differentiating cells, IL-4 or IL-13 was added to the basal medium when cells were first exposed to air; medium with or without IL-4 or IL-13 was refreshed three times a week.

Primary bronchial epithelial cells (PBEC) were obtained from anonymized tumour-free lung tissue obtained at lung resection surgery for lung cancer by enzymatic digestion as described previously [19]. Cells from passage 2 were cultured at an air- liquid interface as described previously [9]. In short, cultures were grown submerged for 4-7 days until they reached confluence, after which they were cultured at an air-liquid interface (ALI) for another 2 weeks in medium containing a high concentration of retinoic acid (15 ng/ml; Lonza, Breda, The Netherlands) to induce mucociliary differentiation. IL-4 or IL-13 (Peprotech, Rocky Hill, NJ) was added during these two weeks of culture at the ALI (see Figure 1). Mucociliary differentiation was usually observed between day 7 and 10 after exposure to air-liquid interface. ALI cultures were maintained for 14 days at 37°C in a humidified atmosphere of 5% CO_2_. The apical side of the epithelial layers was washed with warm PBS three times a week; medium and stimuli were refreshed at the same time. At the end of the 14-day ALI culture period (48 hours after the last addition of stimulus), basal medium (BM) was harvested and frozen until further use. The apical side of the epithelial cultures was incubated with 100 μl of PBS containing 1% N-Acetyl-L-cysteine (Sigma-Aldrich) for 15 minutes at room temperature to dissolve mucus threads; this apical wash (AW) was frozen until further use.

### RNA isolation, reverse transcription and polymerase chain reaction

RNA was isolated at the end of the 14-day ALI culture using the Qiagen RNeasy Minikit (Qiagen, Valencia, CA) in combination with the Qiagen RNase-Free DNase Set (Qiagen, Valencia, CA); an additional DNAseI treatment was performed to remove residual chromosomal DNA. Subsequently, RNA was cleaned using a tenth volume 3 M Sodium Acetate (pH 5.2), 2 volumes ice cold ethanol absolute and precipitation overnight at -20°C. The RNA concentration and purity were determined by measuring optical density measurements using the NanoDrop ND-1000 UV-Vis Spectrophotometer (Nanodrop Technologies, Wilmington, DE). Reverse transcription of 500 ng of total RNA was performed using 200 U of Moloney murine leukemia virus reverse transcriptase (Invitrogen); 0.5 mM of dATP, dCTP, dGTP, dTTP (Invitrogen); 0.5 μg of oligo(dT)_15 _primer (Promega, Madison, WI); RNasin RNase inhibitor 40 U (Promega); 10 mM dithiothreitol (Invitrogen); 50 mM Tris-HCl (pH 8.3); 75 mM KCl; and 3.0 mM MgCl_2 _in a total volume of 20 μl.

PCR was performed at 95°C for 4 minutes followed by 25 or 35 cycles 15 seconds denaturing, 15 seconds annealing and 30 seconds amplification using 0.5 pmol gene specific primers (table 1); nucleotide mix (Invitrogen, Breda, The Netherlands) in a final concentration of 0.08 mM each; 1 U of GoTaq Flexi DNA polymerase (Promega, Madison, WI); 5 μl of 5× Green GoTaq Flexi reaction buffer (Promega) and different concentrations of MgCl_2 _in a final volume of 25 μl. β-actin was used as a reference gene. Products were visualised using a 1.5% agarose gel containing ethidium bromide.

**Table 1:** Primers used for RT-PCR

*Name*	*Primer sequence*	*Melting temperature (°C)*	*MgCl_2 _(mM)*	*Product size (base pairs)*
β-actin	F:GGAAGGCTGGAAGAGTGC R:CTACAATGAGCTGCGTGTGG	56	2	528
hCAP18/LL-37	F:GGCTCCTTTGACATCAGTT R:CTGTCCTGGGTACAAGATTCC	55	2.5	150
hBD-2	F:ATCAGCCATGAGGGTCTTG R:GCAGCATTTTGTTCCAGG	62	2	190
hBD-3	F:AGCCTAGCAGCTATGAGGATC R:CTTCGGCAGCATTTTGCGCCA	62	2	206
MUC5AC	F:ATTTTTTCCCCACTCCTGATG R:AAGACAACCCACTCCCAACC	58	2	146
MUC2	F:GACGCACTGTATCATCAAACG R:AGGATGGTCGTGTTGATGCG	58	2	409
CBE-1	F:GGTCAGAGCTTGAGGTAGCATGTG R:GAGTTGTAACAGCACACTGCATTC	60	3	395
Tektin	F:AGCTGGCTGATCATCTGGCCAAG R:GGATTTCCTCATCTGCATACACAG	60	2	358

### Quantitative real time Polymerase Chain Reaction

Quantitative real time PCR was performed on a MyiQ Single-Color Real-Time PCR detection system with IQ5 software (Bio-Rad Laboratories, Veenendaal, The Netherlands) using IQ SYBR Green supermix containing 3 mM MgCl_2 _(Bio-Rad) and gene specific primers (table 2). Samples were analysed in triplicate and each PCR included a no-template control and a standard curve in duplicate, made from a sample containing the appropriate cDNA. PCR was performed at 95°C for 4 minutes followed by 40 cycles of 15 seconds denaturing, 15 seconds annealing and 30 seconds amplification; fluorescence was measured after each amplification step. A melt curve analysis was performed to determine the primer specificity and the absence of primer dimers. Bio-Rad IQ5 software was used to calculate relative mRNA quantity using the relative standard curve method and to normalize the expression of the genes of interest to both reference genes (GAPDH and β-actin).

**Table 2:** Primers used for quantitative real time PCR

*Name*	*Primer Sequence*	*Melting Temperature (°C)*	*Product size (base pairs)*
β-actin	F: TGCGTGACATTAAGGAGAAG R: TGAAGGTAGTTTCGTGGATG	63	213
GAPDH	F: TTCCAGGAGCGAGATCCCT R: CACCCATGACGAACATGGG	62	259
hCAP18/LL-37	F: TCATTG CCCAGGTCCTCAG R: TCCCCATACACCGCTTCAC	64.3	249
MUC5AC	F: CCTTCGACGGACAGAGCTAC R: TCTCGGTGACAACACGAAAG	63.5	111
SLPI	F: CCAGGGAAGAAGAGATGTTG R: CCTCCATATGGCAGGAATC	61	226
Elafin	F: CCGCTGCTTGAAAGATACTG R: GAATGGGAGGAAGAATGGAC	59.2	177

### MUC5AC and hCAP18/LL-37 dot blot

To determine whether MUC5AC protein and hCAP18/LL-37 protein were released by ALI cultures, apical washes of the cultures were diluted and spotted on a methanol-preincubated polyvinylidene-difluoride (PVDF)-membrane using a Bio-Dot Microfiltration apparatus (Bio-Rad). For MUC5AC, non-specific binding sites were blocked with PBS/5% w/v skim milk (Fluka, Buchs, Switzerland) overnight at 4°C, and subsequently the membrane was incubated with 2 μg/ml of mouse-anti-MUC5AC (clone 45M1; Labvision Neomarkers, Fremont, CA) in PBS/5% (w/v) skim milk for 1 h at room temperature. For hCAP18/LL-37, a similar protocol was used. After spotting the samples, non-specific binding sites were blocked using PBS/5% (v/v) heat-inactivated newborn calf serum (NBCS; Gibco, Grand Island, NY)/5% (v/v) skim milk (Fluka, Buchs, Switzerland) for 2 h at room temperature. Subsequently, the membrane was incubated overnight at 4°C with monoclonal anti-LL-37 antibody 1.1C12 (2 μg/ml) in PBS/5% (v/v) heat-inactivated NBCS/5% (w/v) skim milk. A HRP-conjugated goat-anti-mouse IgG (subclasses 1+2a+2b+3antibody) (Jackson ImmunoResearch Laboratory, Bar Harbor, ME) was used as a secondary antibody for MUC5AC and a HRP-conjugated goat-anti-mouse Ig antibody (Dako) for the LL-37 dot blot and detected using ECL Western Blotting substrate (Pierce, Rockford, IL). Densitometry was performed using Totallab image analysis software (Nonlinear Dynamics, Newcastle upon Tyne, UK).

### hCAP18/LL-37 Western Blot

Basal media, apical washes and cell lysates were harvested after 14 days of culture. Four ml of BM and 200 μl of AW were pooled per stimulus, concentrated using Oasis HLB 1cc extraction cartridges (Waters Chromotography, Etten-Leur, The Netherlands), and subjected to SDS-PAGE on a 16.5% Tris-Tricine gel as described earlier [20]. After protein separation, proteins were blotted onto PVDF for 1.5 h and subsequently blocked with PBS/5% (v/v) heat-inactivated NBCS/5% (w/v) skim milk. The monoclonal mouse anti-hCAP18/LL-37 clone 1.1.C12 (diluted 1:1000) in combination with goat-anti-mouse-HRP (Dako; diluted 1:1000) or a polyclonal rabbit-anti-hCAP18/LL-37 (Innovagen; diluted 1:200) together with swine-anti-rabbit-HRP (Dako: diluted 1:1000) in combination with ECL were used to detect hCAP18/LL-37. Synthetic LL-37 (0.5 ng) and a nasal secretion sample were used as positive controls for LL-37 and hCAP18 respectively. Kaleidoscope polypeptide standard (Bio-Rad) was used as a molecular weight marker.

### Measurement of protein levels of antimicrobial peptides hCAP18/LL-37, hBD-2, hBD-3 and the antimicrobial protease inhibitors SLPI and elafin

All proteins were measured using well-established sandwich ELISAs. hCAP18/LL-37 and elafin/SKALP levels were measured using commercially available kits (Hycult biotechnology, Uden, The Netherlands). hBD-3 levels were measured using the human beta-defensin-3 ELISA kit (Phoenix Pharmaceuticals, Karlsruhe, Germany). SLPI release was measured using a sandwich ELISA as described earlier [21], and hBD-2 levels were measured as previously described [22].

### Antimicrobial assay

The antimicrobial assay was adapted from Diamond *et al*. [23]. ALI cultures of PBEC were established. Two days prior to harvesting, medium was replaced by antibiotics-free growth medium and the last stimulation with IL-4 and IL-13 was performed. Apical wash was harvested using 70 μl of 10 mM sodium phosphate buffer pH 7.4 and stored at -20°C. 250 colony forming units (CFU) of mid-log phase *Pseudomonas aeruginosa *strain PAO1 (BAA-47, American Type Culture Collection, Rockville, MD, USA) were inoculated into AW (42.5 μl bacteria added to 7.5 μl of AW) and left shaking for 3 hours at 37°C. The number of CFU/ml was determined by plating serial dilutions on LB agar plates; colonies were counted after overnight incubation at 37°C.

### Morphology of air-liquid interface cultures

The morphology of the cultures was assessed using light microscopy and transmission electron microscopy (TEM). At the end of the cultures, cells were fixed using a solution of 1% paraformaldehyde (Merck)/1.5% glutaraldehyde (BDH Laboratory Supplies, Poole, UK)/0.1 M sodium cacodylate buffer (pH 7.4, Merck) for 20 minutes at room temperature, washed in cacodylate buffer, post-fixed using 1% osmium tetraoxide (BDH) in 0.1 M sodium cacodylate buffer for 10 minutes, rinsed in distilled H_2_O and dehydrated in ethanol. Subsequently the filters were embedded in 1:1 epon (PolyBed, Polysciences, Germany)/ethanol absolute for at least one hour, followed by a 2:1 epon/ethanol absolute for another hour. Filters were then embedded in pure epon in a mould and the epon was allowed to polymerize at 60°C for at least 24 hours. One micron sections were stained using 1% Toluidin Blue and viewed with a Leica DM/RB microscope and sequential ultra-thin sections were examined in a JEOL JEM-1011 electron microscope with digital photography using a MegaView III camera.

### Immunofluorescence staining of MUC5AC in air-liquid interface cultures

ALI cultures were fixed in 1% w/v paraformaldehyde (Merck) in PBS. Subsequently, the filters were blocked with PBS/1% w/v BSA/0.05% v/v Tween-20 for 30 minutes at 4°C, washed with PBS and incubated with methanol for 20 minutes at 4°C. After washing with PBS, primary antibodies were incubated overnight at 4°C (mouse-anti-MUC5AC clone 45M1 at 0.2 μg/ml); followed by washing with PBS and incubation with Alexa Fluor-labeled secondary antibodies (goat-anti-mouse IgG (H+L)-Alexa Fluor 488 for 2 h at 4°C. After washing with PBS, filters were mounted on glass slides, covered with Vectashield (Vector Laboratories, Burlingame, CA) and a coverglass. Pictures were taken using a confocal Laser Scanning Microscope (Zeiss LSM510; Zeiss, Jena, Germany).

### Statistics

Results are expressed as mean ± SEM. Data were analyzed using the non-parametric Wilcoxon signed ranks test. Differences at p values smaller than 0.05 were considered statistically significant.

## Results

### Two-week exposure of differentiating bronchial epithelial cells to IL-4 or IL-13 enhances hCAP18/LL-37 and hBD-2 mRNA expression as well as hCAP18/LL-37 protein release

**Figure 2:**
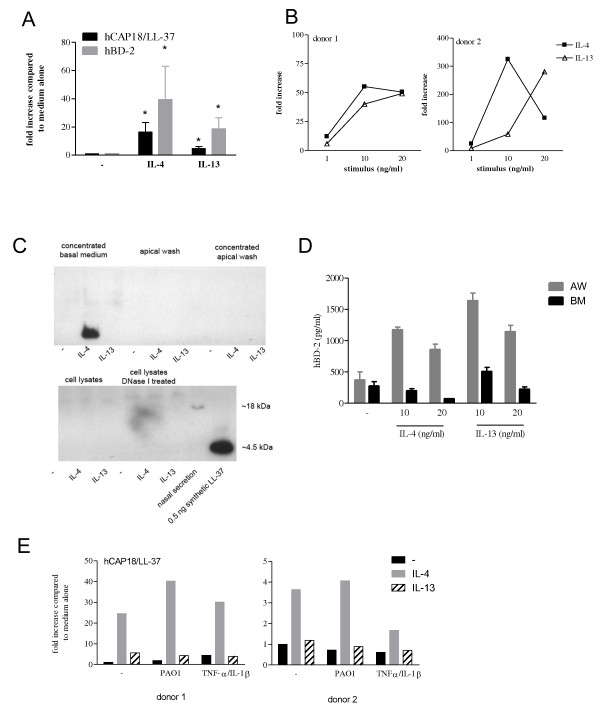
**Two week exposure to IL-4 or IL-13 increases the expression of the antimicrobial peptides hCAP18/LL-37 and hBD-2**. PBEC from various donors were cultured at an ALI for two weeks in the presence of various concentrations of IL-4 or IL-13. (A) Quantitative real time PCR of LL-37 and hBD-2. Data were normalized for expression against two reference genes (β-actin and GAPDH). Data are represented as mean ± SEM of fold increase compared to medium alone for five different donors. * p < 0.05 Wilcoxon signed ranks test. (B) Densitometry results (fold increase compared to medium alone) from the dot blot analysis of hCAP18/LL-37 expression in apical washes of ALI cultures using cells from two donors. (C) Western Blot of LL-37 in cultures treated with medium alone (-), IL-4 (20 ng/ml), or IL-13 (20 ng/ml). From left to right: basal medium concentrated using Oasis cartridges, apical washes, apical washes concentrated using cartridges, cell lysates, cell lysates treated with DNaseI obtained from one culture, and a nasal secretion sample and 0.5 ng of synthetic LL-37 as controls. Similar results were obtained in basal medium from another donor using another antibody specific for hCAP18/LL-37. (D) ELISA for hBD-2 on apical washes and basal medium; mean ± SEM of n = 3 and n = 4 experiments, respectively. (E) Two ALI cultures that were differentiated in the absence or presence of IL-4 or IL-13 for two weeks were subsequently stimulated with 10^8 ^CFU heat-inactivated *P. aeruginosa *PAO1 or with a combination of TNF-α and IL-1β (both at 20 ng/ml) for 24 h. Quantitative real time PCR of hCAP18/LL-37. Data were normalized for expression against two reference genes (β-actin and GAPDH). Two separate experiments are shown; data are represented as fold increase compared to medium alone.

To study how exposure to IL-4 or IL-13 during differentiation of bronchial epithelial cells affects expression of antimicrobial peptides, we exposed PBEC that were cultured at the ALI to various concentrations of these cytokines for 14 days to induce differentiation (see Figure 1). Both IL-4 and IL-13 enhanced mRNA expression of hCAP18/LL-37 and hBD-2 in ALI cultures using cells from five different donors (Figure 2A). hBD-3 mRNA expression was very low in the ALI cultures (data not shown). IL-4 and IL-13 increased hCAP18/LL-37 protein release as assessed by dot blot analysis of apical washes obtained from two ALI cultures using cells from the same two donors (Figure 2B). Using a sandwich ELISA for hCAP18/LL-37 with a lower detection limit of 100 pg/ml, we were not able to detect the protein in apical wash or basal medium. However, using SDS-PAGE and Western Blot analysis, we show that concentrated basal medium of ALI cultures treated with IL-4 contain a protein of approximately 4.5 kDa (Figure 2C), suggesting that hCAP18 is cleaved into a smaller fragment which could either be LL-37 or other peptides known to be cleaved from hCAP18 [24]. hBD-2 protein level in apical washes was increased by both IL-4 and IL-13 (Figure 2D), whereas in basal medium it was not. hBD-3 protein release into apical washes as measured by ELISA was variable: it was undetectable in cultures from two out of six donors; IL-4 induced hBD-3 protein release in cultures of two donors, whereas it decreased hBD-3 protein release in cultures of two other donors (data not shown). We did not assess effects of IL-13 on hBD-3 expression.

These data show that exposure to Th2 cytokines during differentiation increases the mRNA expression and protein release of hCAP18/LL-37 and hBD-2 by bronchial epithelial cells, but has an inconclusive effect on hBD-3 expression. In addition, our data show that hCAP18 is released into the basal medium and is cleaved into a smaller fragment of approximately 4.5 kDa containing an epitope recognized by LL-37 antibodies.

Previously it was shown that pro-inflammatory stimuli and bacteria increase hBD-2 expression in epithelial cells, and the induction was inhibited when Th2 cytokines were added during stimulation. We have now also investigated whether differentiation in the presence of IL-4 or IL-13 affected bacteria- or TNF-α/IL-1β-induced AMP expression. In two experiments we observed that in ALI cultures differentiated in medium alone, PAO1 and TNF-α/IL-1β induced a marked increase in hBD-2 expression, which was reduced (to a varying degree in two donors), when cells were differentiated in the presence of IL-4, indeed suggesting that Th2 cytokines may inhibit hBD-2 expression (data not shown). However, hCAP18/LL-37 expression was increased to a greater extent by IL-4 and IL-13 differentiation than by the pro-inflammatory stimuli suggesting that differentiation of the epithelium is important for induction of hCAP18/LL-37 rather than pro-inflammatory stimuli (Figure 2E).

### Two-week exposure of differentiating epithelial cells to IL-4 or IL-13 does not affect the expression of SLPI and elafin protein release

**Figure 3:**
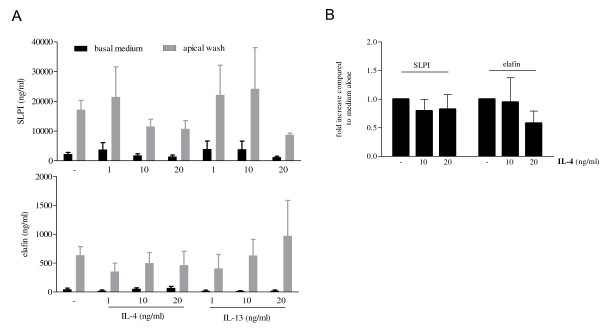
**Two week exposure to IL-4 or IL-13 does not affect the release of the antimicrobial proteinase inhibitors SLPI or elafin**. PBEC from six different donors were cultured at an ALI for 14 days in the presence of 1, 10 or 20 ng/ml of IL-4 or IL-13. (A) Basal medium (black bars) and apical washes (gray bars) were harvested and analyzed for SLPI (upper panel) and elafin (lower panel) protein levels using sandwich ELISA. Data are presented as mean ± SEM from experiments performed in duplicate (- n = 8; IL-4 1 ng/ml n = 3; IL-4 10 ng/ml n = 8; IL-4 20 ng/ml n = 7; IL-13 1 ng/ml n = 3; IL-13 10 ng/ml n = 3; IL-13 20 ng/ml n = 2). (B) Quantitative real time PCR for SLPI and elafin mRNA expression. Data are mean ± SEM of fold increase compared to medium alone for 4 independent experiments. Data were normalized for expression against two reference genes (β-actin and GAPDH).

To study the effect of presence of Th2 cytokines during differentiation on release of the antimicrobial proteinase inhibitors SLPI and elafin, PBEC were cultured at an ALI for 14 days in the absence or presence of various concentrations of IL-4 and IL-13 during differentiation. Basal medium and apical washes were harvested at the end of the culture. No significant differences in SLPI or elafin release in basal medium (BM) or apical wash (AW) were found when IL-4- and IL-13-treated cultures were compared to medium-treated cultures (Figure 3A). In addition, no effects on mRNA expression were found (Figure 3B). These data show that the antimicrobial protease inhibitors SLPI and elafin are not increased during IL-4- or IL-13-induced differentiation of ALI cultures.

### Two-week exposure of differentiating epithelial cells to IL-4 or IL-13 enhances antimicrobial activity in apical wash

**Figure 4:**
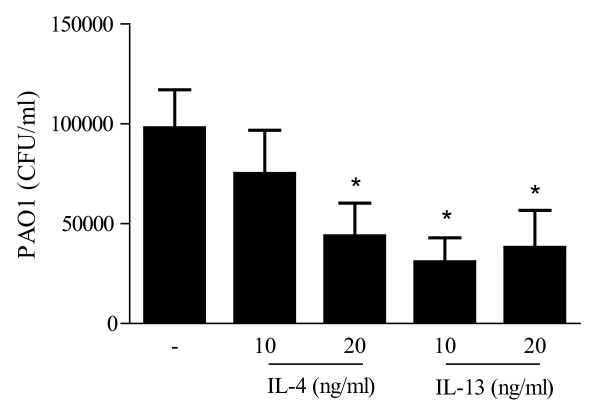
**Two week exposure to IL-4 or IL-13 enhances antimicrobial activity**. Apical wash was harvested using a 10 mM sodium phosphate buffer pH 7.4 and added to 250 colony forming units (CFU) of log phase *P. aeruginosa *PAO1 for 3 h. Bacteria were plated to assess number of CFU. Data represent mean CFU/ml ± SEM using apical washes obtained from six different donors.

To assess whether IL-4- and IL-13-treated ALI cultures displayed antimicrobial activity, we harvested apical wash and measured antimicrobial activity. Growth of *Pseudomonas aeruginosa *was inhibited by apical wash obtained from six separate ALI cultures treated with IL-4 or IL-13 (Figure 4). When compared to phosphate buffer alone, medium-treated ALI cultures also exhibited some antimicrobial activity (data not shown). We did not find any antimicrobial activity in basal medium of the IL-4- and IL-13-treated cultures.

### Two-week exposure of differentiating epithelial cells to IL-4 affects the morphology of the epithelial layer

**Figure 5:**
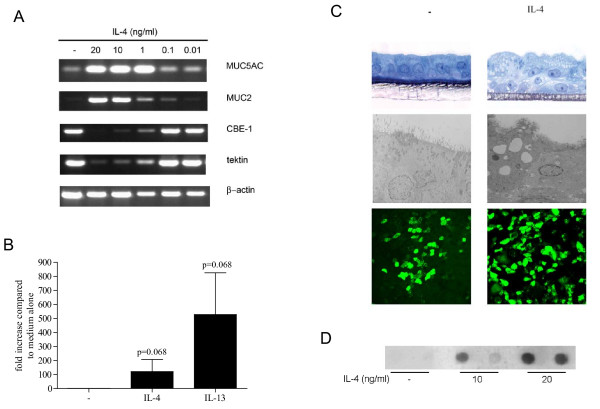
**Two week exposure to IL-4 alters the morphology and expression of markers for goblet cells and ciliated cells**. (A) Expression of mucins and markers of ciliated cells at the mRNA level in ALI cultures exposed to various concentrations of IL-4 for two weeks. (B) QPCR analysis of MUC5AC in ALI cultures differentiated using IL-4 or IL-13. Data were normalized for expression against two reference genes (β-actin and GAPDH). (C) Upper panels: toluidin blue staining of an untreated (left upper panel) and IL-4-treated (10 ng/ml) ALI culture (right upper panel). Middle panels: transmission electron microscope picture of the same cultures. Lower panels: MUC5AC protein expression in an untreated and IL-4-treated ALI culture after staining with a mouse-anti-MUC5AC antibody (clone 45M1) and goat-anti-mouse IgG-Alexa488 as a secondary antibody. (D) MUC5AC dot blot of apical washes of ALI cultures stimulated with 10 or 20 ng/ml of IL-4 of one donor. Increase in MUC5AC protein was observed in five out of six experiments using cells from four different donors.

As it was previously shown that IL-4 and IL-13 affect bronchial epithelial cell differentiation [13], we investigated morphology and markers of ciliated and goblet cells. Also in our cultures, differentiation in the presence of IL-4 affects morphology; a thickened layer containing more goblet cells is formed (Figure 5C). An increase in MUC5AC mRNA expression was observed in IL-4- and IL-13-treated cultures using quantitative real time PCR (Figure 5B), whereas a reduction of FoxJ1 mRNA expression was found in 5 out of 6 donors (data not shown). Reverse transcriptase PCR showed that IL-4 also reduced mRNA expression of tektin and CBE-1 (other markers of ciliated epithelial cells), whereas expression of MUC2 mRNA was enhanced (Figure 5A). MUC5B mRNA expression was not altered by IL-4. Using dot blot analysis, we observed that IL-4 slightly increased MUC5AC protein release in five out of six experiments using cells from four different donors (Figure 5D). These data confirm previous findings [10-13] by showing that also in our cultures, two-week exposure to IL-4 during differentiation alters the morphology of bronchial epithelial cells, increases the production of MUC5AC, and decreases expression of markers of ciliated cells.

### Exposure of already differentiated bronchial epithelial cells to IL-4 does not affect hCAP18/LL-37 expression

Next we assessed whether IL-4 also increases hCAP18/LL-37 expression in already differentiated epithelial layers. To this end, we differentiated bronchial epithelial cells at an ALI for 14 days using retinoic acid to induce mucociliary differentiation; subsequently we stimulated the cells with 10 or 20 ng/ml of IL-4 for 48 hours and assessed mRNA expression for hCAP18/LL-37, SLPI and elafin. In this model, IL-4 did not induce mRNA expression of hCAP18/LL-37 (n = 2, data not shown). These data suggest that the altered differentiation (increase in goblet cells) induced by IL-4 and IL-13 is responsible for the increase in hCAP18/LL-37.

## Discussion

In this study, we investigated whether the presence of Th2 cytokines during mucociliary differentiation of bronchial epithelial cell cultures affects antimicrobial defence. Our results show that two-week exposure of differentiating epithelial cells to the Th2 cytokine IL-4 induces an increase in hCAP18/LL-37 and hBD-2 mRNA and protein expression, whereas levels of the antimicrobial proteinase inhibitors SLPI and elafin were not affected. In addition to IL-4, also exposure to IL-13 during differentiation resulted in an increase of hCAP18/LL-37 and hBD-2 mRNA, as well as increased protein expression in apical washes. However, only in basal medium of IL-4-treated cultures a peptide of approximately 4.5 kDa was detected suggestive of the antimicrobial peptide LL-37. Apical wash obtained from ALI cultures treated with IL-4 or IL-13 exhibited increased antimicrobial activity compared to medium-treated cultures as shown by increased killing of *P. aeruginosa *PAO1 suggesting that increased expression of LL-37 and hBD-2 contribute to the increased antimicrobial activity.

In line with previous findings [12,13], in our experiments IL-4 and IL-13 exposure increased MUC5AC mRNA expression. Using various concentrations of IL-4 we also showed that MUC5AC protein expression was increased and markers of ciliated cells were decreased, suggesting that goblet cell hyperplasia was induced. As we did not observe effects of IL-4 on AMPs expression when added to already-differentiated cultures, perhaps the IL-4- or IL-13-induced changes in differentiation underlie the observed increased expression of antimicrobial peptides and increased antimicrobial activity. In other models of differentiated epithelia, such as a model of human gingiva [25] and in differentiated colonic epithelium [26], increased expression of hCAP18/LL-37 and β-defensins has also been shown, and was suggested to be related to the differentiation status. In our study, hCAP18/LL-37 and hBD-2 expression was induced in cultures that were differentiated in the presence of IL-4 or IL-13 which resulted in an epithelial layer resembling goblet cell hyperplasia in e.g. asthmatic epithelium. In contrast, short-term (48 h) IL-4 treatment of already fully-differentiated cultures (using retinoic acid) did not affect hCAP18/LL-37 mRNA levels. It has been shown by others that 48 h stimulation of mucociliary differentiated epithelial layers with IL-4 or IL-13 did not result in increased release of mediators (GM-CSF and TGF-β2), contrary to what occurs in submerged cultures [27]. These data are in concordance with our data on the lack of effect of short term stimulation on hCAP18/LL-37 expression by mucociliary differentiated epithelial layers. Taken together these data suggest that increased hCAP18/LL-37 and hBD-2 expression in airway epithelial cells is perhaps related to the altered differentiation of the epithelial cell layer. However, we have not yet been able to identify which cell type in our ALI cultures is responsible for the production of the antimicrobial peptides.

In contrast to other studies showing a reduction in PAO1- or TNFα/IL-1β-induced hBD-2 expression by Th2 cytokines, we now show that differentiation in the presence of IL-4 or IL-13 increases hCAP18/LL-37 and hBD-2 expression in the absence of bacterial or pro-inflammatory stimuli. In separate experiments we observed that although IL-4 and IL-13 increase hBD-2 expression in our culture model, also when IL-4- or IL-13-differentiated cultures were subsequently stimulated with PAO1 or TNF-α/IL-1β, hBD-2 mRNA induction by these stimuli was slightly reduced. hCAP18/LL-37 expression, however, was not affected by 24 h stimulation with PAO1 or TNFα/IL-1β whereas differentiation in the presence of IL-4 or IL-13 increased the expression. These data suggest that differentiation in the presence of Th2 cytokines alone results in increased expression of AMPs, but can also hamper induction of AMP expression by bacterial and other pro-inflammatory stimuli. Whether the same effects exist when Th1 or pro-inflammatory cytokines are also present during differentiation remains the question.

Using an antimicrobial activity assay, we have now shown that presence of IL-4 or IL-13 during differentiation leads to increased antimicrobial activity. Previously it was shown that airway surface fluid has a complex composition of various mediators including many antimicrobial peptides [28], which may additively or synergistically enhance antimicrobial activity. We measured low levels of hBD-2 (1-1.5 ng/ml after IL-4 or IL-13) and were unable to detect LL-37 using a sandwich ELISA with a 100 ng/ml lower detection limit. It has been reported that 0.16 μg/ml of hBD-2 kills 50% of *E. coli *[28], whereas clinical isolates of *S. aureus *were very resistant [14]. In addition it was shown 5 μg/ml of hBD-2 was needed to kill 50% of *P. aeruginosa *(clinical isolate PA-O) [29], whereas 16 μg/ml of synthetic LL-37 was needed to kill *P. aeruginosa *ATCC 27853 [30]. Since the levels of hBD-2 and LL-37 that we detected were much lower, it is unlikely that the antimicrobial activity of apical washes obtained from IL-4- or IL-13-treated cultures is solely due to the activity of LL-37 or hBD-2 alone. However, it has been shown that low (subinhibitory) concentrations of LL-37, lactoferrin and SLPI synergize with lysozyme to kill E. coli [28]. It has been previously shown that ALI cultures of bronchial epithelial cells express small amounts of lysozyme and lactoferrin [31]. Using IL-9, another Th2 cytokine, in differentiating epithelial cells also results in an increase in goblet cells and lysozyme [32]. In addition, it has been shown that the combination of LL-37 and hBD-2 kills more *S. aureus *than hBD-2 [14]. Therefore, perhaps the increased antimicrobial activity of the apical washes of our IL-4- and IL-13-treated cultures is due to synergistic or additive effects of LL-37 or hBD-2 with lysozyme or other compounds since the expression of both LL-37 and hBD-2 is increased in these cultures compared to medium-treated cultures. In the future, neutralizing various AMPs in apical washes may provide an answer to the question which AMPs are responsible for the increased antimicrobial activity of IL-4- and IL-13-treated cultures observed.

Our study has some limitations. We were not able to detect hCAP18/LL-37 expression in washes obtained from the apical side of the cultures using a sandwich ELISA and SDS-PAGE followed by Western Blot, whereas this apical wash did display antimicrobial activity. As it is known that LL-37 can bind to mucus and DNA [33,34], perhaps epitopes are masked due to these interactions which leads to the detection problems found in the aforementioned assays. Another possibility is that levels of hCAP18/LL-37 were below the detection limits of our assays. In addition, we were only able to detect hCAP18/LL-37 in the basal medium of IL-4-treated cultures and not in IL-13-treated cultures, whereas both cytokines increased mRNA and protein expression assessed by dot blot analysis. We do not think this is due to the fact that IL-13 is less potent in inducing antimicrobial substances as it was equipotent if not more potent in the antimicrobial assay and the induction of hBD-2 protein release. Since we also did not detect hCAP18/LL-37 in basal medium using a higher amount of IL-13 (100 ng/ml) during differentiation (unpublished data), possibly IL-13 does not induce sufficient secretion of hCAP18/LL-37 to the basal side, but only to the apical side.

Which clinical implications do our results have? The increase in AMP expression after differentiation in the presence of Th2 cytokines may contribute to the first line of defence, which is necessary until inflammatory cells capable of dealing with the microbes are present at the site of invasion. It is known that neutrophils are a potent source of hCAP18/LL-37 and neutrophil defensins, and that these cells probably produce greater amounts of these AMPs than epithelial cells. We have not been able to measure quantifiable amounts of hCAP18/LL-37 using the sandwich ELISA suggesting that very low amounts (<100 pg/ml) of hCAP18/LL-37 are present in our cultures or that this method is not suitable for detection of this AMP in our cultures. However, as the apical washes of the IL-4- and IL-13-treated cultures displayed increased antimicrobial activity, this confirms that the airway epithelium contributes to the antimicrobial defence. In addition, the increase in hCAP18/LL-37 and hBD-2 may result in increased activation of the adaptive immune response, since it is known that these AMPs can act as chemoattractants for phagocytes and dendritic cells, and LL-37 can affect DC differentiation [35].

In conclusion, differentiation of epithelium in the presence of Th2 cytokines IL-4 and IL-13 results in an altered epithelial layer that not only produces more mucus, but also more hCAP18/LL-37 and hBD-2, and displays increased antimicrobial activity.

## Competing interests

SZ is sponsored by a research grant from Boehringer Ingelheim Pharma GmbH&Co. KG, Biberach, Germany. DN, JS, RV, MS, FP and SW have no competing interests. The Department of Pulmonology, and thereby KR as head of the department and PH as staff member, has received grants from AltanaPharma, Amgen, Centocor, Novartis, Bayer, AstraZeneca, Pfizer, MSD, Exhale Therapeutics, Boehringer Ingelheim, Roche and GSK. KR and PH have been consulting, participated in Advisory Board Meetings and received lecture fees from AstraZeneca, Amgen, Boehringer, Centocor, Chiesi Pharmaceuticals, Pfizer, Novartis, AltanaPharma, MSD, and GSK.

## Authors' contributions

SZ was involved in conception and design of the study, performing experiments, analyzing and interpreting data and drafted and revised the manuscript. DN and JS performed experiments and were involved in analyzing and interpreting data. MS and RV substantially assisted in performing all of the cell cultures. FP assisted in experiments involving histological and immunofluorescence techniques. SW and KR were involved in conception of the study, and critically revised the manuscript. PH was involved in conception and design of the study, interpretation of data and drafting and revision of the manuscript. All authors have read and approved the manuscript.

## References

[B1] Van Wetering S van der Linden AC van Sterkenburg MA de Boer WI Kuijpers AL Schalkwijk J Regulation of SLPI and elafin release from bronchial epithelial cells by neutrophil defensins Am J Physiol Lung Cell Mol Physiol 2000 278 L51 L58 10645890 10.1152/ajplung.2000.278.1.L51

[B2] Williams SE Brown TI Roghanian A Sallenave JM SLPI and elafin: one glove, many fingers Clin Sci (Lond) 2006 110 21 35 10.1042/CS20050115 16336202

[B3] Hiemstra PS Maassen RJ Stolk J Heinzel-Wieland R Steffens GJ Dijkman JH Antibacterial activity of antileukoprotease Infect Immun 1996 64 4520 4524 8890201 10.1128/iai.64.11.4520-4524.1996 PMC174407

[B4] Simpson AJ Wallace WA Marsden ME Govan JR Porteous DJ Haslett C Adenoviral augmentation of elafin protects the lung against acute injury mediated by activated neutrophils and bacterial infection J Immunol 2001 167 1778 1786 11466403 10.4049/jimmunol.167.3.1778

[B5] Sallenave JM Antimicrobial activity of antiproteinases Biochem Soc Trans 2002 30 111 115 12023836 10.1042/

[B6] McNeely TB Shugars DC Rosendahl M Tucker C Eisenberg SP Wahl SM Inhibition of human immunodeficiency virus type 1 infectivity by secretory leukocyte protease inhibitor occurs prior to viral reverse transcription Blood 1997 90 1141 1149 9242546

[B7] Del Prete GF De Carli M D'Elios MM Maestrelli P Ricci M Fabbri L Allergen exposure induces the activation of allergen-specific Th2 cells in the airway mucosa of patients with allergic respiratory disorders Eur J Immunol 1993 23 1445 1449 10.1002/eji.1830230707 8100770

[B8] Robinson DS Hamid Q Ying S Tsicopoulos A Barkans J Bentley AM Predominant TH2-like bronchoalveolar T-lymphocyte population in atopic asthma N Engl J Med 1992 326 298 304 10.1056/NEJM199201303260504 1530827

[B9] Van Wetering S Zuyderduyn S Ninaber DK van Sterkenburg MA Rabe KF Hiemstra PS Epithelial differentiation is a determinant in the production of eotaxin-2 and -3 by bronchial epithelial cells in response to IL-4 and IL-13 Mol Immunol 2007 44 803 811 10.1016/j.molimm.2006.04.008 16740309

[B10] Zhu Z Homer RJ Wang Z Chen Q Geba GP Wang J Pulmonary expression of interleukin-13 causes inflammation, mucus hypersecretion, subepithelial fibrosis, physiologic abnormalities, and eotaxin production J Clin Invest 1999 103 779 788 10.1172/JCI5909 10079098 PMC408149

[B11] Laoukili J Perret E Willems T Minty A Parthoens E Houcine O IL-13 alters mucociliary differentiation and ciliary beating of human respiratory epithelial cells J Clin Invest 2001 108 1817 1824 11748265 10.1172/JCI13557 PMC209466

[B12] Yoshisue H Puddicombe SM Wilson SJ Haitchi HM Powell RM Wilson DI Characterization of ciliated bronchial epithelium 1, a ciliated cell-associated gene induced during mucociliary differentiation Am J Respir Cell Mol Biol 2004 31 491 500 10.1165/rcmb.2004-0050OC 15242845

[B13] Atherton HC Jones G Danahay H IL-13-induced changes in the goblet cell density of human bronchial epithelial cell cultures: MAP kinase and phosphatidylinositol 3-kinase regulation Am J Physiol Lung Cell Mol Physiol 2003 285 L730 L739 12794003 10.1152/ajplung.00089.2003

[B14] Ong PY Ohtake T Brandt C Strickland I Boguniewicz M Ganz T Endogenous antimicrobial peptides and skin infections in atopic dermatitis N Engl J Med 2002 347 1151 1160 10.1056/NEJMoa021481 12374875

[B15] Nomura I Goleva E Howell MD Hamid QA Ong PY Hall CF Cytokine milieu of atopic dermatitis, as compared to psoriasis, skin prevents induction of innate immune response genes J Immunol 2003 171 3262 3269 12960356 10.4049/jimmunol.171.6.3262

[B16] Albanesi C Fairchild HR Madonna S Scarponi C De Pita O Leung DY IL-4 and IL-13 negatively regulate TNF-alpha- and IFN-gamma-induced beta-defensin expression through STAT-6, suppressor of cytokine signaling (SOCS)-1, and SOCS-3 J Immunol 2007 179 984 992 17617590 10.4049/jimmunol.179.2.984

[B17] Howell MD Gallo RL Boguniewicz M Jones JF Wong C Streib JE Cytokine milieu of atopic dermatitis skin subverts the innate immune response to vaccinia virus Immunity 2006 24 341 348 10.1016/j.immuni.2006.02.006 16546102

[B18] Beisswenger C Kandler K Hess C Garn H Felgentreff K Wegmann M Allergic airway inflammation inhibits pulmonary antibacterial host defense J Immunol 2006 177 1833 1837 16849494 10.4049/jimmunol.177.3.1833

[B19] Van Wetering S van der Linden AC van Sterkenburg MA Rabe KF Schalkwijk J Hiemstra PS Regulation of secretory leukocyte proteinase inhibitor (SLPI) production by human bronchial epithelial cells: increase of cell-associated SLPI by neutrophil elastase J Investig Med 2000 48 359 366 10979241

[B20] Tjabringa GS Vos JB Olthuis D Ninaber DK Rabe KF Schalkwijk J Host defense effector molecules in mucosal secretions FEMS Immunol Med Microbiol 2005 45 151 158 10.1016/j.femsim.2005.03.004 16051067

[B21] Wingens M van Bergen BH Hiemstra PS Meis JF Vlijmen-Willems IM Zeeuwen PL Induction of SLPI (ALP/HUSI-I) in epidermal keratinocytes J Invest Dermatol 1998 111 996 1002 10.1046/j.1523-1747.1998.00425.x 9856807

[B22] Proud D Sanders SP Wiehler S Human rhinovirus infection induces airway epithelial cell production of human beta-defensin 2 both in vitro and in vivo J Immunol 2004 172 4637 4645 15034083 10.4049/jimmunol.172.7.4637

[B23] Diamond G Yim S Rigo I McMahon L Measuring antimicrobial peptide activity on epithelial surfaces in cell culture Methods Mol Biol 2010 618 371 382 10.1007/978-1-60761-594-1_23 20094876 PMC2811320

[B24] Murakami M Lopez-Garcia B Braff M Dorschner RA Gallo RL Postsecretory processing generates multiple cathelicidins for enhanced topical antimicrobial defense J Immunol 2004 172 3070 3077 14978112 10.4049/jimmunol.172.5.3070

[B25] Peyret-Lacombe A Duplan H Watts M Charveron M Brunel G Antimicrobial peptide modulation in a differentiated reconstructed gingival epithelium Cell Tissue Res 2007 328 85 95 10.1007/s00441-006-0344-8 17216197

[B26] Hase K Eckmann L Leopard JD Varki N Kagnoff MF Cell differentiation is a key determinant of cathelicidin LL-37/human cationic antimicrobial protein 18 expression by human colon epithelium Infect Immun 2002 70 953 963 10.1128/IAI.70.2.953-963.2002 11796631 PMC127717

[B27] Kikuchi T Shively JD Foley JS Drazen JM Tschumperlin DJ Differentiation-dependent responsiveness of bronchial epithelial cells to IL-4/13 stimulation Am J Physiol Lung Cell Mol Physiol 2004 287 L119 L126 10.1152/ajplung.00365.2003 15020299

[B28] Singh PK Tack BF McCray PB Welsh MJ Synergistic and additive killing by antimicrobial factors found in human airway surface liquid Am J Physiol Lung Cell Mol Physiol 2000 279 L799 L805 11053013 10.1152/ajplung.2000.279.5.L799

[B29] Harder J Meyer-Hoffert U Teran LM Schwichtenberg L Bartels J Maune S Mucoid Pseudomonas aeruginosa, TNF-alpha, and IL-1beta, but Not IL-6, Induce Human beta -Defensin-2 in Respiratory Epithelia Am J Respir Cell Mol Biol 2000 22 714 721 10837369 10.1165/ajrcmb.22.6.4023

[B30] Bals R Wang X Zasloff M Wilson JM The peptide antibiotic LL-37/hCAP-18 is expressed in epithelia of the human lung where it has broad antimicrobial activity at the airway surface Proc Natl Acad Sci USA 1998 95 9541 9546 10.1073/pnas.95.16.9541 9689116 PMC21374

[B31] Gray TE Guzman K Davis CW Abdullah LH Nettesheim P Mucociliary differentiation of serially passaged normal human tracheobronchial epithelial cells Am J Respir Cell Mol Biol 1996 14 104 112 8534481 10.1165/ajrcmb.14.1.8534481

[B32] Vermeer PD Harson R Einwalter LA Moninger T Zabner J Interleukin-9 induces goblet cell hyperplasia during repair of human airway epithelia Am J Respir Cell Mol Biol 2003 28 286 295 10.1165/rcmb.4887 12594054

[B33] Felgentreff K Beisswenger C Griese M Gulder T Bringmann G Bals R The antimicrobial peptide cathelicidin interacts with airway mucus Peptides 2006 27 3100 3106 10.1016/j.peptides.2006.07.018 16963160

[B34] Bucki R Byfield FJ Janmey PA Release of the antimicrobial peptide LL-37 from DNA/F-actin bundles in cystic fibrosis sputum Eur Respir J 2007 29 624 632 10.1183/09031936.00080806 17215317

[B35] Davidson DJ Currie AJ Reid GS Bowdish DM MacDonald KL Ma RC The cationic antimicrobial peptide LL-37 modulates dendritic cell differentiation and dendritic cell-induced T cell polarization J Immunol 2004 172 1146 1156 14707090 10.4049/jimmunol.172.2.1146

